# Preoperative Evaluation of Axillary Lymph Nodes in Malignant Breast Lesions with Ultrasonography and Histopathologic Correlation

**DOI:** 10.5334/jbr-btr.899

**Published:** 2016-04-15

**Authors:** Nurdan Fidan, Emine Ozturk, Cuneyt Yucesoy, Baki Hekimoglu

**Affiliations:** 1 Hitit University Training and Research Hospital, TR; 2 Ankara Diskapı Training and Research Hospital, Ankara, TR

**Keywords:** Axillary ultrasonography, Axillary lymph node, Breast cancer, Metastasis

## Abstract

**Purpose:**

Our aim is to define the sonographic criteria for assessing involved axillary nodes and to evaluate the accuracy of axillary ultrasound in the staging workup of individuals with breast cancer.

**Materials and Methods:**

Thirty-five patients with breast cancer were prospectively evaluated with preoperative ultrasonography (US) to determine the presence of axillary lymph node metastasis. We determined whether there was axillary lymph node metastasis after axillary lymph node dissection or sentinel lymph node biopsy. If metastasis was found, the number of metastatic lymph nodes was recorded and compared with preoperative axillary US findings using histopathological evaluation as a reference.

**Results:**

Metastatic lymph node detection in sonographic evaluation was associated with echogenic hilus obliteration, complete hypoechoic or anechoic appearance of lymph nodes, and asymmetric/nodal or diffuse cortical thickening greater than 3.8 mm. The overall sensitivity, specificity, positive predictive value, and negative predictive value of US were calculated as (20/22) 91 percent, (10/13) 77 percent, (20/23) 87 percent, and (10/12) 83 percent, respectively.

**Conclusion:**

Ultrasonography examination is a valuable method for evaluating the axilla in newly diagnosed breast cancer patients and provides valuable information for planning proper breast cancer management.

## Introduction

Lymph node involvement and tumor size are the most important factors in the prognosis of breast cancer and remain crucial for individual treatment decisions [1,2,3]. Historically, axillary lymph node dissection (ALND) has been accepted as a reference standard for the diagnosis of lymph node involvement, but because of side effects such as lymphedema, paresthesia, and restriction of movement, sentinel lymph node biopsy (SLNB) has replaced ALND as the primary staging procedure in many centers [4,5]. SLNB is less invasive and is associated with a lower morbidity.

It has been reported in previous studies that if there is no sign of preoperative axillary lymph node metastasis in patients, SLNB can be applied. Axillary dissection is not necessary in cases of either negative SLNB or in the presence of isolated tumor cells or micrometastases. But if there is at least one lymph node with the presence of cytologically demonstrated metastasis, ALND or neoadjuvant chemotherapy can be done without performing SLNB [5,6,7,8,9]. Thus, identifying the appropriate selection criteria for SLNB becomes increasingly important, and the necessity of evaluation of the axillary region in the preoperative period with noninvasive methods has arisen.

Mammography is inadequate to detect metastatic lymph nodes because the entire axillary region cannot be completely evaluated with this method. Positron emission tomography combined with computed tomography (PET/CT) and magnetic resonance imaging (MRI) do not have a place in routine staging because of their higher costs and possible side effects [10,11]. Ultrasonography (US) is the most widely used imaging method to detect axillary lymph node metastasis and for characterization of lymph nodes [1,12].

New ultrasound techniques, including contrast-enhanced ultrasound (CEUS) and US elastography, have been deployed for lymph node evaluation. CEUS provides detailed visualization of the vascularity of lymph nodes and may thus be helpful in differentiating between benign and malignant nodes [13]. With regard to axillary staging, only a small number of studies have been published, with very preliminary results [14,15]. US elastography is strongly dependent on the experience of the examiner and cannot be recommended for routine clinical use.

In this study, we present the results of preoperative assessment with US of axillary lymph nodes in terms of metastasis, metastatic lymph node detection rates, and sensitivity and specificity of US compared with postoperative histopathological results.

## Materials and Methods

### Patient Population

This single-center study was conducted between 2010 and 2011 in the radiology clinic of our hospital. We obtained institutional review board approval before commencing this prospective study. Patients were evaluated with unilateral axillary ultrasonography (AUS) and had category 5 or 6 breast lesions according to BI-RADS classification with mammography and ultrasonography. Lymph nodes were evaluated according to morphological criteria. A total of 35 patients with breast carcinoma were included in the study. Thirty-two patients underwent mastectomy or breast-conserving surgery with ALND. We compared our US findings with ALND results in these patients. ALND was not performed in 3 patients who underwent breast-conserving surgery because SLNB frozen results were reported as reactive lymph node during their operations. These patients were included in the patient group with negative postoperative axillary lymph node metastasis due to the preoperative imaging studies being negative.

Age of the patients; menopausal status; family history; hormone use; physical examination findings; mammography and US findings; localization of malignant masses in the breast; the number and dimensions of the lymph nodes with reactive, suspicious, and metastatic characteristics detected in the axillary region with US; postoperative mass histology, size, and grade; the presence of axillary lymph node metastasis, the number and size; and type of breast surgery performed were recorded.

### Ultrasound Techniques and Characterization of Lymph Nodes

Ultrasonographic evaluation was performed by a single radiologist with five years of experience in the field of ultrasonography using a GE Logiq S6 with a 7–12 MHz high-frequency probe or Powervision 6000 SSA-370A with a 6–11 MHz high-frequency linear probe. Level I, II, and III lymph nodes in the axillary region supraclavicular lymph nodes and internal mammary lymph nodes on the side of malignant breast lesions were evaluated and recorded. Each node was classified sonographically according to the cortical morphological findings into three groups, including reactive (benign), suspicious, and metastatic lymph nodes (lymphadenopathy-LAP).

In the *benign group*, hyperechoic lymph nodes had an invisible and thin diffuse hypoechoic cortex (< 3 mm) with significant echogenic “fatty” hilus (Figures 1a and 1b). In the *suspicious group*, lymph nodes had asymmetric focal (Figures 2a and 2b) or diffuse cortical thickening (> 3 mm) (Figures 3a and 3b), lobulated and more hypoechoic cortex compared to subcutaneous fat, with significant echogenic hilusa and distorted hilus. In the *metastatic group*, the lymph nodes had obliterated and unselected hilus and were completely hypoechoic or anechoic in appearance (Table 1). Cortical thickness of the lymph nodes was measured in the longitudinal section. If the lymph node showed asymmetric thickening, the thickest portion was measured. The number of lymph nodes, whether there was axillary lymph node metastasis, and if there was, the number of metastatic lymph nodes and the size of the largest metastatic lymph node, which was learned from the postoperative pathology report, were recorded and compared with the preoperative AUS findings. The size of the lymph nodes was measured sonographically; however, the size criteria was not used alone in the classification.

**Figure 1a:**
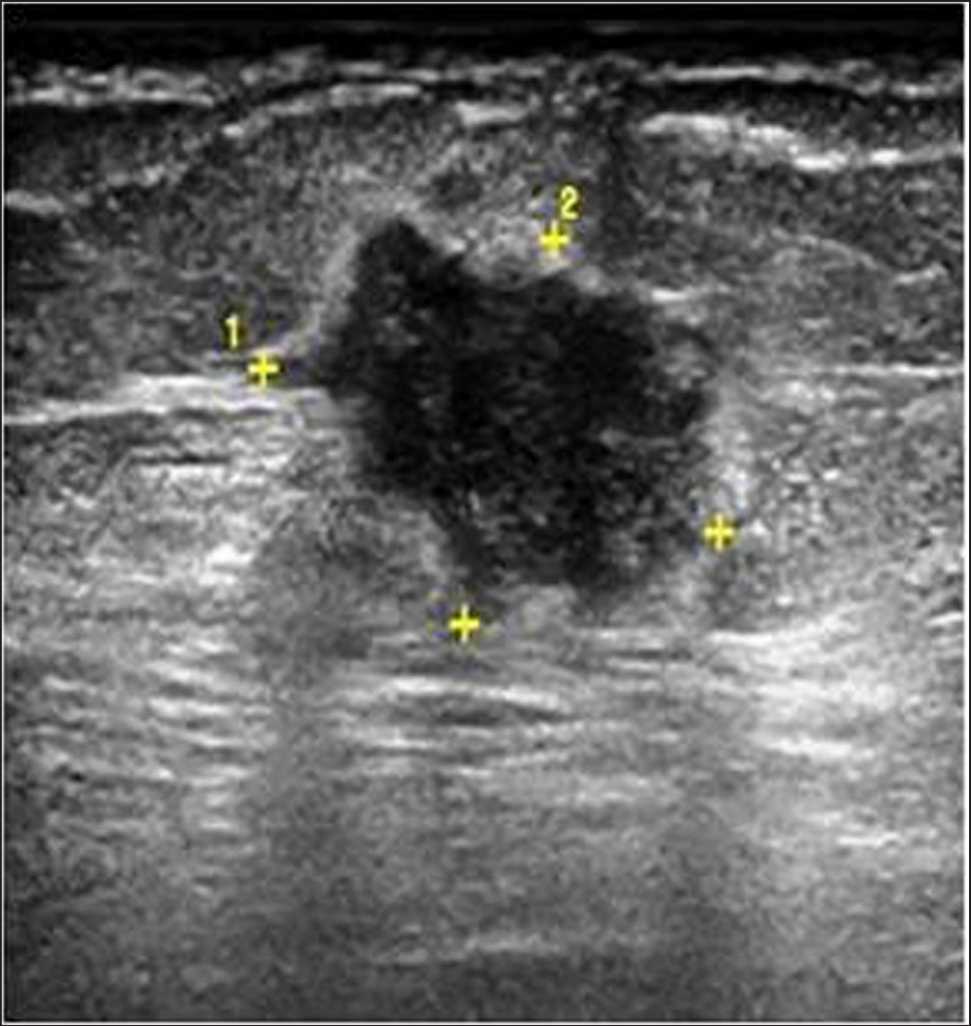
A 69-year-old woman with invasive ductal carcinoma. Breast US shows irregularly shaped and hypoechoic solid lesions which are highly suspicious for malignancy.

**Figure 1b:**
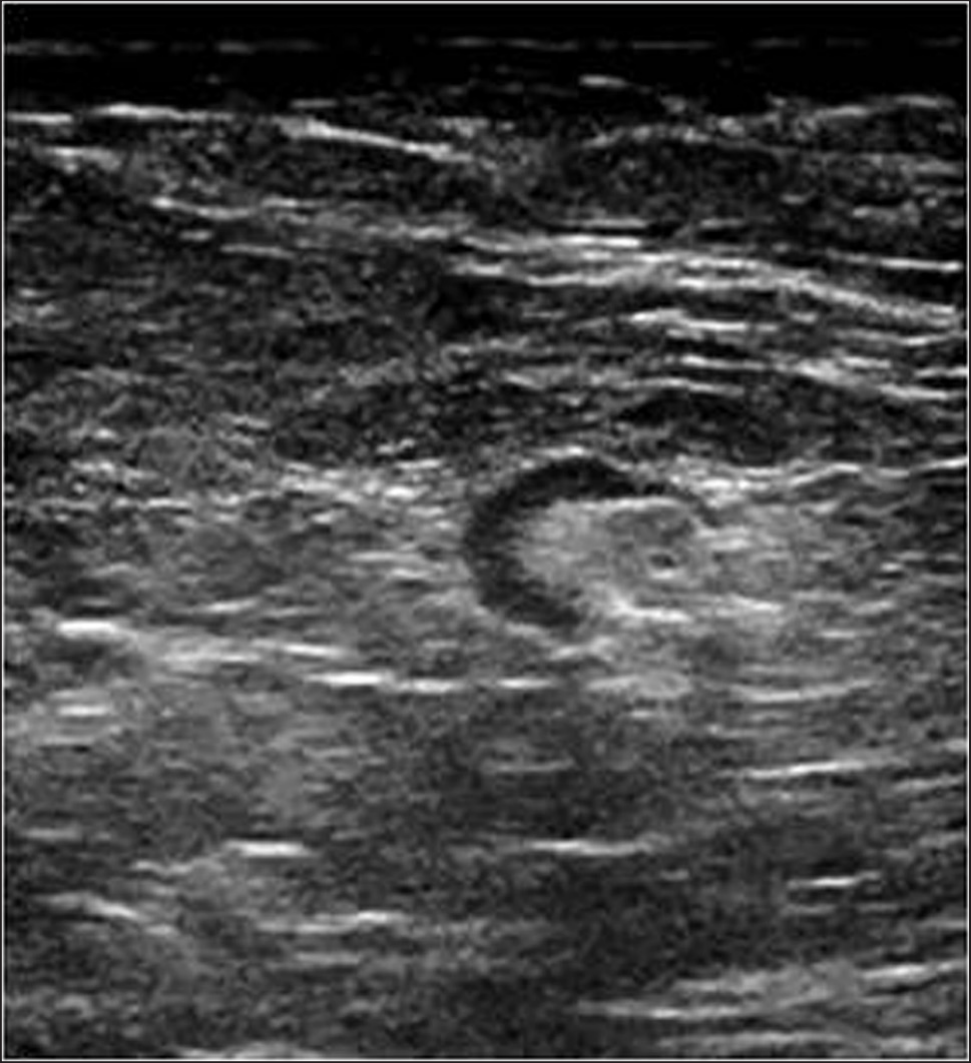
Ultrasound image of the same patient’s left axillary region at Level 1 shows a benign lymph node with diffuse thin cortex and wide echogenic hilum (metastatic involvement was not found histopathologically).

**Figure 2a:**
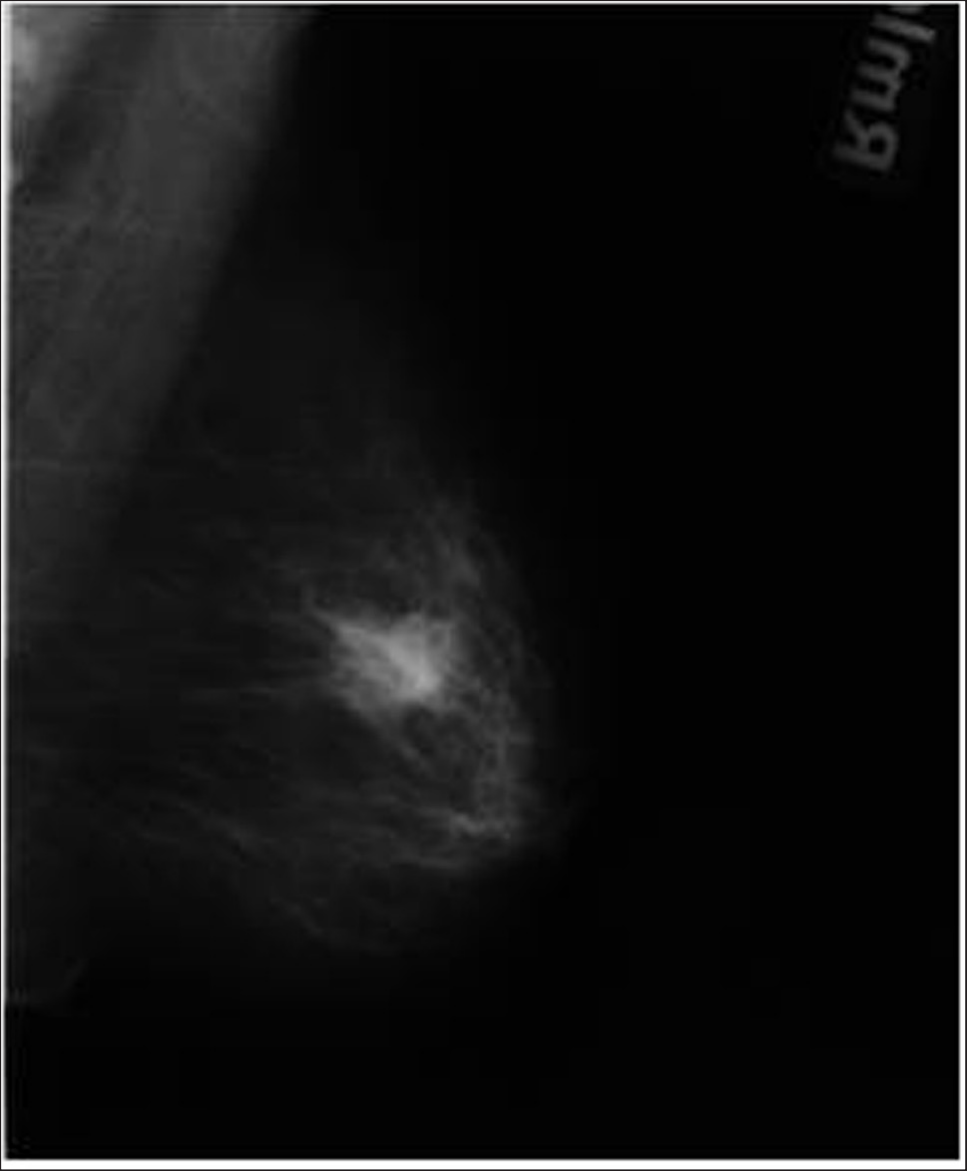
A 52-year-old woman with invasive ductal carcinoma. The mammographic image shows a spiculated dense mass.

**Figure 2b:**
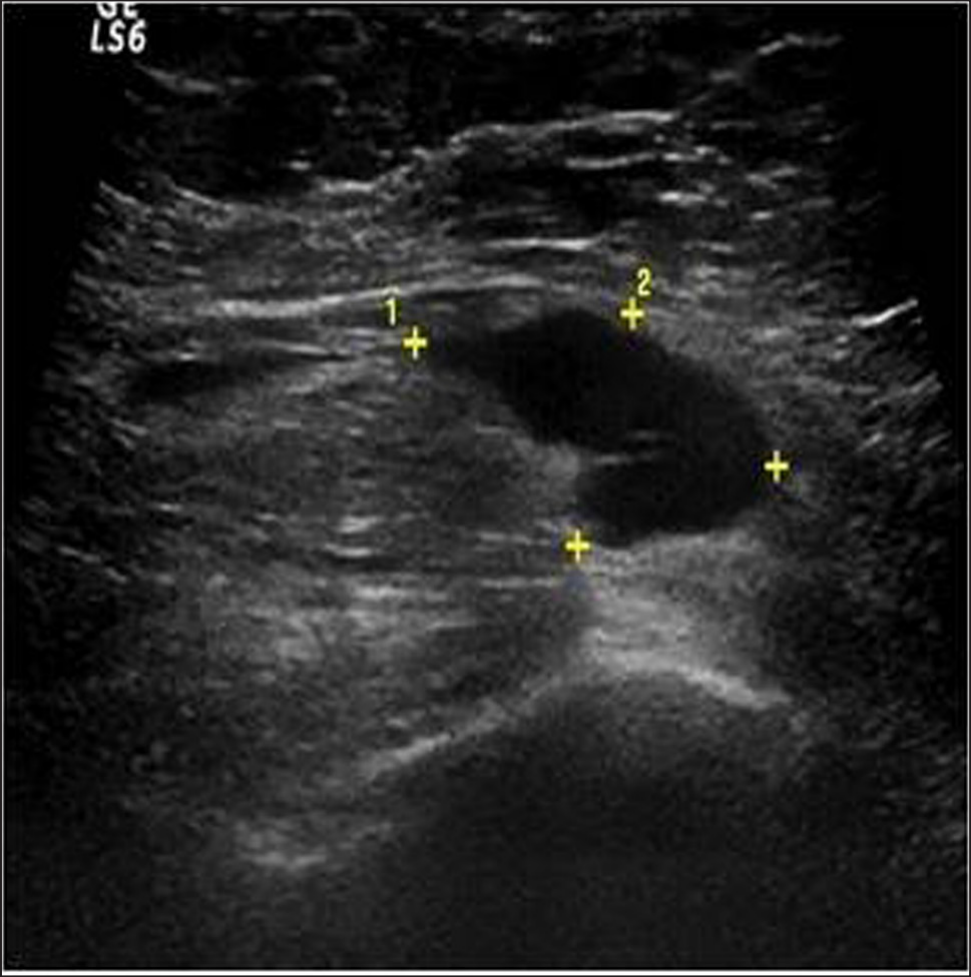
Ultrasound image of the same patient’s right axillary region at Level 1 demonstrates suspicious lymph node with asymmetric cortical thickening and markedly hypoechoic cortex (metastatic involvement was found histopathologically).

**Figure 3a:**
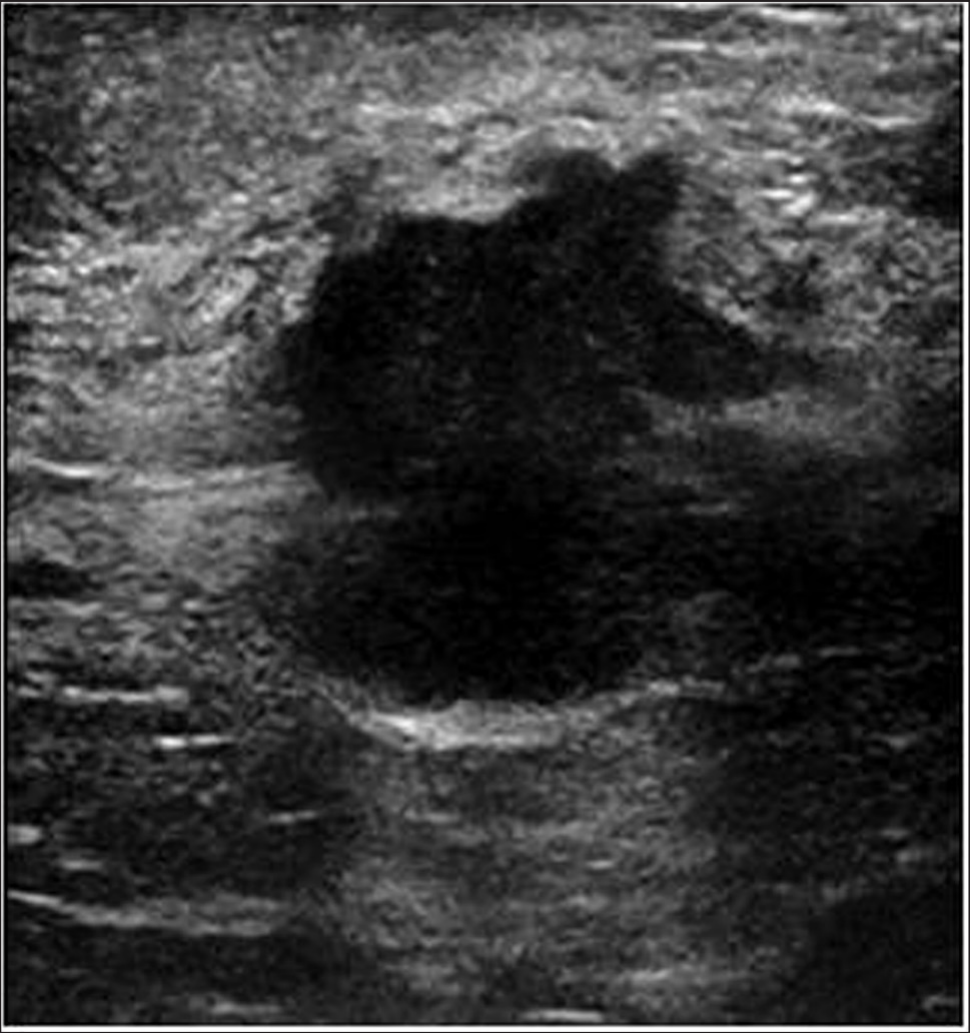
A 55-year-old woman with invasive ductal carcinoma. Breast US shows irregularly shaped and hypoechoic solid lesions which are highly suspicious for malignancy.

**Figure 3b:**
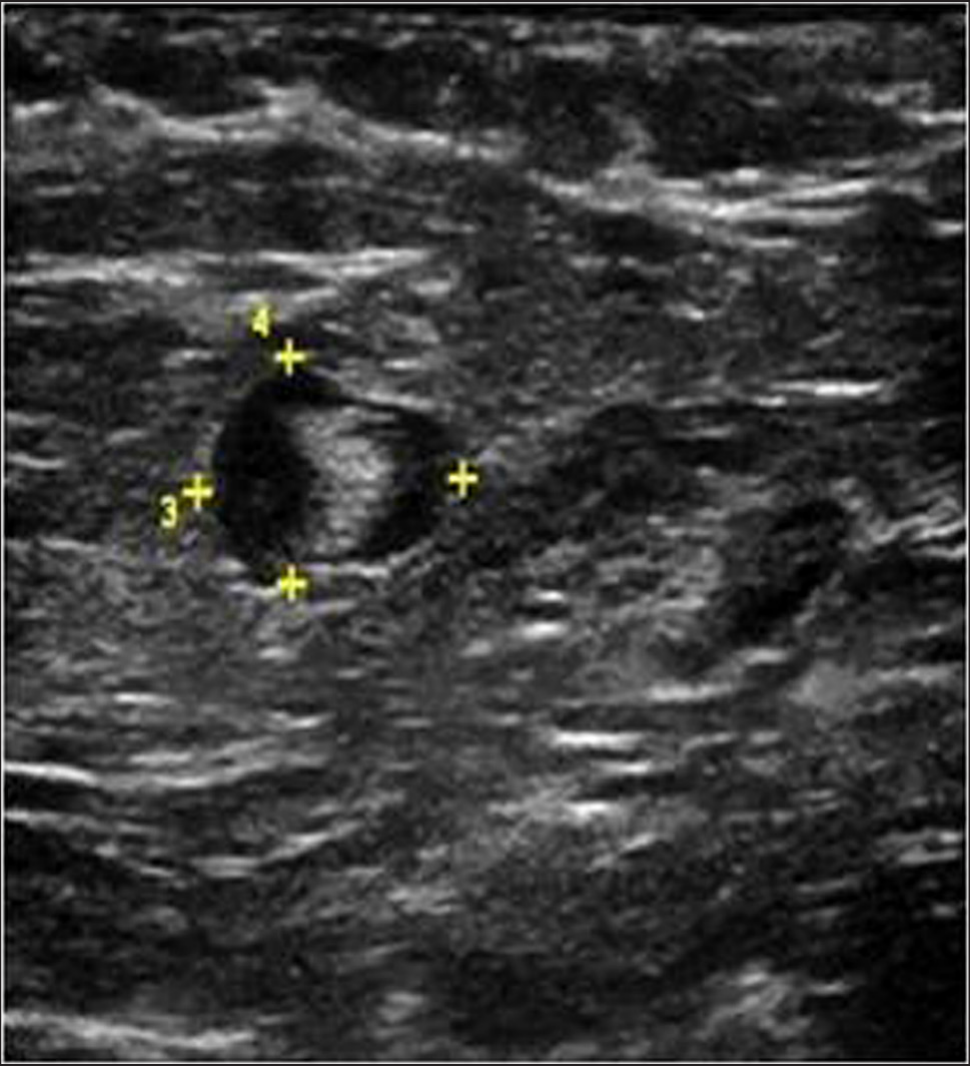
Ultrasound image of the same patient’s left axillary region at Level 1 shows suspicious lymph node with diffuse cortical thickening and hypoechoic cortex (metastatic involvement was found histopathologically).

**Table 1:** US Features of Axillary Lymph Nodes Used to Assess Susupicion for Malignancy.

Parameters	benign	suspicious	metastatic

Cortex	absent/thin	> 3 mm	> 3 mm
	diffuse/thin	focally asymmetric lobulated	thick
		thick	
		markedly hypoechoic*	hypo/anechoic
Fatty hilum	central	eccentric distortioned	completely replaced

*Hypoechoic cortex was defined as being more hypoechoic than subcutaneous tissue.

### Statistical Analysis

Patients were divided into five groups depending on preoperative AUS findings and postoperative histopathological findings. The presence of at least one lymph node with metastatic character was accepted as a positive finding, and the absence was accepted as a negative finding. The presence of at least one lymph node with suspicious character was considered a suspicious finding. Histopathologically, determining at least one lymph node metastasis in the axilla was considered a positive finding, and if no lymph nodes contained metastasis, this was considered a negative finding. Groups are shown in Table 2.

**Table 2:** Patient Groups.

Groups	Number of patients (n:35)	Preoperative axillary US findings	Postoperative nodal metastasis	Postoperative mean size of the primary tumor (mm)	Postoperative mean size of the lymph nodes (mm)

Group 1	10	negative	negative	15,6	–
Group 2	11	positive	positive	27	16,9
Group 3	9	suspicious	positive	26,6	11,5
Group 4	3	suspicious	negative	36	–
Group 5	2	negative	positive	26	8

For statistical analysis, SPSS for Windows (version 16.0) software was used. Descriptive statistics are reported as mean (minimum-maximum) values for continuous variables and as frequency with percentages for categorical variables. Group comparisons for continuous variables were tested using the Mann-Whitney U or Wilcoxon Z test as appropriate. Comparisons of categorical variables were evaluated by a chi-square test.

## Results

The average age of the patients included in the study was 55.6 (31–82) years. Other demographic data of patients and tumor characteristics are summarized in Table 3. A total of 131 lymph nodes of 35 patients were evaluated by US.

**Table 3:** Patient Demographics and Tumor Characteristics.

Mean age of the patients (years)	55,6
Mean size of the primary tumor (mm)	25,1
Number of the masses according to their localization in the breast
Upper outer quadrant	20
Upper inner quadrant	6
Lower outer quadrant	3
Lower inner quadrant	6
Clinical manifestation
Palpable breast mass	26
Pain	4
Asymptomatic	2
Other*	3
Axillary palpation finding
Positive	4
Negative	31
Family history
Positive	2
Negative	33
Hormone use
Positive	2
Negative	33
Primary tumor type
Ductal	32
Ductal + lobular	1
Ductal + mucinous	1
Metaplastic	1
T stage (according to AJCC classification)
T1	12
T2	21
T3	2
T4	0
Tumor grade
G1	7
G2	18
G3	10

* Refers to nipple discharge or nipple retraction. Source: American Joint Commitee on Cancer (AJCC).

In the first group, 10 patients were examined, and 47 lymph nodes were considered benign. In this group, the average cortical thickness was found to be 2.1 mm (0.8–3 mm). Metastatic involvement was not found histopathologically in any of these patients.

In the second group, 11 patients were examined, and 38 and 10 lymph nodes were considered as metastatic and suspicious, respectively. The cortical thickness was not measured due to the hilus of metastatic lymph nodes being obliterated. The mean cortical thickness of 10 suspicious lymph nodes was found to be 3.4 mm (3–4.5 mm). Metastatic involvement was found histopathologically in all of these patients, and the number of positive lymph nodes in preoperative ultrasonography (mean 4.36) and the number of postoperative LAP (mean 5.9) were statistically correlated (p > 0.05).

In the third group, 9 patients were examined, and 9 and 16 lymph nodes were considered as suspicious and benign, respectively. The mean cortical thickness of the suspicious and benign lymph nodes was found to be 4.4 mm (3–7.4 mm) and 2 mm (1.3–2.9 mm), respectively. Metastatic involvement was found histopathologically in all of these patients, and the number of positive lymph nodes in preoperative ultrasonography (mean 1) and the number of postoperative LAP (mean 2.6) were statistically correlated (p > 0.05).

In the fourth group, 3 patients were examined, and 3 and 5 lymph nodes were considered as suspicious and benign, respectively. In this group, three suspicious lymph nodes showing 3 mm and 4 mm diffuse thickening were present. The mean cortical thickness of the suspicious and benign lymph nodes was 3.3 mm (3–4 mm) and 2.6 mm (2–2.9 mm), respectively. Metastatic involvement was not found histopathologically in three patients in this group.

In the fifth group, 2 patients were examined, and 3 lymph nodes were considered benign. The mean cortical thickness in this group was 2.1 mm (1.3–2.6 mm). Metastatic involvement was found histopathologically in two patients in this group.

While positive findings were present on preoperative AUS in 23 patients (65.7%), 12 (34.3%) patients had negative findings. Postoperative axillary lymph node metastasis was positive in 22 (62.9%) and negative in 13 (37.1%) out of 35 patients. Comparing postoperative histopathological confirmation of lymph node metastasis, the sensitivity, specificity, positive predictive value, and negative predictive value of US were calculated as (20/22) 91 percent, (10/13) 77 percent, (20/23) 87 percent, and (10/12) 83 percent, respectively (Table 4).

**Table 4:** Diagnostic Values of Axillary US According to Postoperative Lymph Node Metastasis.

			Postoperative nodal metastasis		Total	Sensitivity	Specificity	Positive predictive value	Negative predictive value

			absent	present					

Preoperative positive US findings	absent	n	10	2	12	0,91	0,77	0,87	0,83
		%	83,3%	16,7%	100,0%				
	present	n	3	20	23				
		%	13,0%	87,0%	100,0%				
Total		n	13	22	35				
		%	37,1%	62,9%	100,0%				

In addition, the average size of primary breast tumors in US for all groups was 23.6 mm (8–65 mm), and the histopathological average size was 25.1 mm (7–52 mm) with a correlation between them (p > 0.05).

## Discussion

Ultrasonography in the evaluation of axilla is the first-choice method of investigation that may be selected because it is easily accessible, noninvasive, and has the ability to guide biopsy procedures. Cortical morphological changes in lymph nodes with metastatic involvement can be assessed by ultrasound as well as the size, contour, and edge sharpness of the lymph node.

Primary tumor size was reported to be one of the most important factors affecting axillary lymph node metastasis [2,3]. In all groups (1–5 groups), histopathological tumor size was correlated with the primary tumor size that we measured with US (p > 0.05). These results indicate the high reliability of primary tumor size measurements with US.

In previous studies, lymph node size or morphological findings or both of them were used together as US criteria. In recent studies, the morphological criteria used in US in detecting metastatic lymph nodes emphasized that totally replaced or eccentric hilus of the lymph node and hypoechoic cortex were the most important morphological US criteria. It was reported that the sensitivity and specificity of US were between 48 percent and 99 percent [2,3,6,9,16,17]. Bedi et al. [16] have measured the size of the lymph nodes in their study and emphasized that the size of benign and malignant lymph nodes were similar, with cortical morphological findings and hypoechoic cortex being more important than lymph node size. We also measured the size of lymph nodes in our study, although we used morphological criteria, not size criteria.

Lymph nodes were detected with a benign character in AUS in 10 of 12 patients (group 1), but histopathological metastatic involvement was not found. The other 2 patients (group 5) with benign lymph node features seen in US were reported to have histopathological metastatic involvement. In these patients, the size of the primary tumor was close to the other groups (groups 2 and 3) with metastatic lymph nodes reported in histopathologic evaluation. However, the average size of metastatic lymph nodes was 8 mm in histopathologic evaluation, and the average histopathological size of metastatic lymph nodes was smaller than in groups 2 and 3 (Table 2). We believe that metastatic lymph nodes were not detected in these 2 patients due to the number of histopathologically metastatic lymph nodes being less, that they were small in size, and the lack of obvious morphological changes in the metastatic lymph nodes. It was reported in previous studies that the sensitivity of US decreased if the number of positive lymph nodes was less than three and there was a decrease in lymph node size [18].

Lymph nodes with suspicious character were detected with AUS in 9 of 12 patients (group 3) with histologic metastatic involvement being found. In 3 of 12 patients (group 4), histopathological metastatic involvement was not determined. The average cortical thickness in the fourth group was lower than the second and third groups where involvement was reported histopathologically and cortical thickening was diffuse. Although diffuse cortical thickening over 3 mm was reported as suspicious criteria in the literature, we believe that 3 mm diffuse cortical thickening is an insufficient morphological criteria alone; focal cortical thickening and contour lobulation are more valuable findings than diffuse thickening in metastasis assessment. Bedi et al. [16] concluded that asymmetric cortical thickness and contour lobulation were more important morphological findings.

In our study, the average cortical thickness was calculated to be 3.8 mm (3–7.4 mm) in suspicious lymph nodes, and 3.8 mm or greater values were detected to be important for metastatic involvement. The average cortical thickness was found to be higher than in previous studies. We think that this situation was caused by the cortical thickness in our patients mostly being a focal feature, and the evaluation of the averages was performed with diffuse cortical thickening.

Lymph nodes were detected with metastatic character with AUS in all patients (group 2) where histopathological metastatic involvement was present, and we saw in our study that echogenic hilus obliteration and complete hypoechoic or anechoic appearance were the most important criteria in determining metastatic lymph nodes.

In this study, the sensitivity of US in the detection of metastatic lymph nodes was calculated to be 91 percent, which was higher than previous similar studies. We believe that this situation may be associated with our detailed prospective anatomical examination of lymph node groups in the axillary region to find lymph node metastasis when we detected a possible malignant mass in the breast.

The small number of our patients and not applying FNA for suspicious lymph nodes by considering clinician preference are the limitations of our study. Although AUS is a method with high sensitivity and specificity for the detection of lymph node metastases, it does not rule out metastasis. It may direct to SLNB cases that are considered to be negative in AUS and ALND can be avoided in the SLNB-negative cases. When metastatic character lymph nodes are observed on AUS, they may be directed to ALND without application of SLNB by providing a correlation with US-guided FNA.

In conclusion, if criteria are taken into account in patients with breast cancer, such as detailed evaluation of the axillary region and echogenic hilus obliteration or eccentric placement, complete anechoic or hypoechoic appearance of the lymph node, and asymmetric cortical thickening, metastatic lymph nodes can be identified by ultrasonography with high sensitivity and positive predictive value. Axillary US may guide the radiologist to carry out an FNA to rule out metastases before deciding whether to perform SLNB.

## Competing Interests

The authors declare that they have no competing interests.
